# Trends in Depression and Its Association With Gallstones: Evidence From NHANES With Exploration of Mediating Pathways

**DOI:** 10.1155/da/1800832

**Published:** 2026-09-22

**Authors:** Enyang He, Yaoqun Wang, Guilin Nie, Qingyan Kong, Yu Teng, Di Zeng, Jiaqi Yang, Xinming Li, Fei Liu, Geng Liu, Bei Li, Nansheng Cheng

**Affiliations:** ^1^ Division of Biliary Surgery, Department of General Surgery, West China Hospital, Sichuan University, Chengdu 610041, Sichuan, China, cd120.com; ^2^ Research Center for Biliary Diseases, West China Hospital, Sichuan University, Chengdu 610041, Sichuan, China, scu.edu.cn; ^3^ Department of Gastrointestinal Surgery, Hebei General Hospital, Shijiazhuang, Hebei, China, hebmu.edu.cn

## Abstract

**Objective:**

This study aimed to analyze long‐term trends (2005–2023) in the prevalence of depression among US adults and to specifically investigate the association between depression and gallstone disease.

**Methods:**

Data from multiple cycles of the National Health and Nutrition Examination Survey (NHANES) were utilized. Depression was assessed using the patient health questionnaire‐9 (PHQ‐9), and gallstone status was determined by self‐reported physician diagnosis. Weighted logistic regression models were employed to evaluate the association between depression and gallstones, with multivariable‐adjusted models controlling for potential confounders. Furthermore, dose–response relationship analysis and mediation analysis were conducted.

**Results:**

Between 2005 and 2023, the prevalence of depression among US adults demonstrated a significant increasing trend. In the 2017–2023 data, depression was significantly associated with higher odds of gallstones (odds ratio [OR] = 1.61, 95% confidence interval [CI]: 1.13–2.29). Dose–response analysis revealed a positive association between increasing PHQ‐9 scores and gallstone presence (*p* for trend <0.001). Mediation analyses suggested that smoking and obesity explained ~6.3% and 6%, respectively, of the total observed association between depression and gallstones.

**Conclusion:**

This study highlights a rising trend in the prevalence of depression and provides evidence for an association between depression and gallstone disease. The association appears to be partially mediated by smoking and body mass index (BMI). These findings underscore the clinical importance of considering gallstone screening and implementing integrated health management strategies for individuals with depression, while further longitudinal studies are warranted to clarify the temporal direction of this relationship.

## 1. Introduction

Gallstone disease is a prevalent global gastrointestinal disorder with an incidence rate of 0.47 per 100 person‐years and a prevalence of 6.1% in adults. It not only causes severe biliary colic but is also the primary indication for cholecystectomy, imposing a substantial burden on healthcare systems and significant economic costs [1–3]. Gallstones are classified into two distinct categories based on their composition: cholesterol gallstones, which represent the predominant entity, and bilirubin stones [4, 5].

Dysfunction of the gallbladder or other parts of the bile‐secretion pathway can result in gallstone formation. The extant literature suggests a correlation between gallstones and a number of factors, including age, gender, female physiological status, obesity, cardiovascular disease, microbiome, sugar metabolism, and various environmental exposures [4, 6, 7]. However, beyond these metabolic and mechanical factors, the role of psychosocial factors in the pathogenesis of gallstones remains under‐explored.

Depression is a common mental disorder whose impact extends far beyond the emotional domain, manifesting as a systemic illness. Extensive research has established that depression is closely associated with multiple biological factors, including genetic predisposition, neurotransmitter systems, brain‐derived neurotrophic factor (BDNF), hyperactivity of the hypothalamic–pituitary–adrenal (HPA) axis, and inflammatory states [8, 9]. The pathophysiological alterations associated with depression are further linked to severe physical health comorbidities, including cardiovascular disease and obesity [10, 11].

It is proposed that the scientific hypothesis, based on the evidence presented, is as follows: depression may increase the risk of gallstone disease through multiple pathways. The following mechanisms have been identified as potential candidates: (1) Lifestyle pathways: Depression is frequently associated with altered appetite, reduced physical activity [10], and unhealthy dietary patterns [9], which may lead to obesity and dyslipidaemia; (2) Neuroendocrine pathways: Hyperactivation of the hypothalamic–pituitary–adrenal axis elevates cortisol levels, thereby impairing gallbladder motility and altering bile composition [12, 13]; and (3) Inflammatory pathways: The chronic inflammatory state associated with depression may promote cholesterol nucleation and induce inflammation in gallbladder tissue [14–16].

Despite the existence of studies indicating a possible correlation between depressive symptoms and gallstone disease [17], there is an absence of substantial evidence from large‐scale, nationally representative populations. Utilizing the NHANES database, a high‐quality resource, this study seeks to innovatively address the following research question: The objective of this study is threefold: firstly, to characterize the long‐term trends in the prevalence of depression among US adults since 2005; secondly, to rigorously examine whether depression is an independent risk factor for gallstones in a large, nationally representative sample; and thirdly, to provide insights for potential interventions targeted at specific high‐risk populations.

## 2. Methods

### 2.1. Study Participants

All data utilized in this study were sourced from the publicly available NHANES database(https://www.cdc.gov/nchs/nhanes/). Data from the 2005 to 2023 survey cycles were used for the analysis of depression, and data from the 2017 to 2023 cycles were used for the analysis of gallstones. The difference in analysis periods is attributed to variable availability in the NHANES database: the depression variables DPQ010–DPQ090 were available for all cycles spanning 2005–2023, supporting the long‐term trend analysis of depression, while the gallstone variable MCQ550 was only collected beginning in 2017 and missing in earlier cycles. For each survey cycle, demographic data were extracted from the DEMO files, information pertaining to depression and gallstones was obtained from interview records, and physical measurements were acquired from examination data. The study population comprised individuals who met the following criteria: (i) participants aged ≥18 years with survey data on race, gender, and the ratio of family income to poverty; (ii) participants for whom interview records are available; and (iii) participants who had undergone physical measurements.

### 2.2. Definition of Depression

Depressive symptoms were assessed using the patient health questionnaire (PHQ‐9). This instrument evaluates the frequency of depressive symptoms over the preceding 2 weeks, with each item scored from 0 to 3. The total score (range: 0–27) was calculated by summing the responses to all nine items (Table S1). In line with established literature [18], which reports a sensitivity of 74% and a specificity of 91% for a cut‐off score of 10, participants with a PHQ‐9 score ≥10 were defined as having depression in this study.

### 2.3. Definition of Gallstones

The presence of gallstones was ascertained by a self‐reported response to the medical condition questionnaire. Specifically, participants were identified as having gallstones if they provided an affirmative response (“Yes”) to the question: “Has a doctor or other health professional ever told you that you had gallstones?” Those responding “No” were considered not to have gallstones.

### 2.4. Ascertainment of Covariates

We adjusted for the following covariates: demographic characteristics (age, sex, and race), socioeconomic status (education level, poverty–income ratio [PIR]), and behavioral and clinical factors (alcohol use, smoking, hypertension, hypercholesterolemia, diabetes, and BMI). The PIR, a measure of socioeconomic status, was derived by dividing the total annual family income by the official poverty guideline for the survey year. The presence of hypertension, hypercholesterolemia, and diabetes was determined by a positive response to the question, “Has a doctor or other health professional ever told you that you have [condition]?” Alcohol drinking was defined as consuming alcoholic beverages more than once a month over the past 12 months [19]. Smoking status was categorized based on the lifetime consumption of at least 100 cigarettes [20].

### 2.5. Mediation Analysis

Causal mediation analysis was conducted using the “mediation” package (version 4.5.1) [21], with the objective of decomposing the total effect into direct and indirect effects via mediation. To enhance the robustness of the assessment, 1000 bootstrap samples were employed, and the average causal mediation effect, average direct effect, and total effect were reported. Furthermore, the proportion and statistical significance of the mediation effect are presented.

### 2.6. Statistical Analysis

To account for the complex, multistage probability sampling design of NHANES, appropriate sampling weights were applied in all analyses. The 2‐year interview weight (WTINT2YR) was used for the epidemiological analysis of depression. For the association analysis between depression and gallstones, the 2‐year mobile examination center weight (WTMEC2YR) was employed. All weighted analyses were conducted using the “survey” package in R software (version 4.3.3). A two‐sided *p*‐value of less than 0.05 was considered statistically significant for all statistical tests.

## 3. Results

### 3.1. Trends in Depression Prevalence

**Figure 1:**
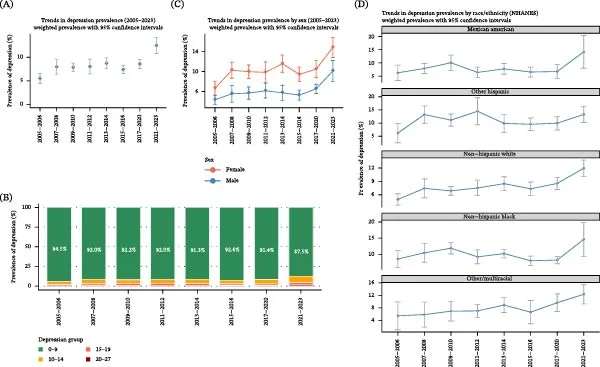
Trends in depression prevalence in the United States. (A) The line chart illustrates shows trends in depression prevalence from 2005 to 2023. (B) The line charts respectively illustrate the prevalence rates of depression between male and female. (C) The proportions in different PHQ‐9 score groups are shown in the bar charts. (D) This line chart shows how prevalent depression is among different ethnic groups.

A total of 88,429 participants from eight NHANES survey cycles (2005–2023) were initially considered. After excluding individuals aged below 18 years (*n* = 34,296) and applying further quality controls, 38,447 participants were included in the final analysis for trend assessment. As illustrated in Figure 1, the prevalence of depression among US adults exhibited a fluctuating but overall upward trend from the 2005 to 2006 cycle to the most recent 2021–2023 cycle. A pronounced surge was observed after 2020, coinciding with the COVID‐19 pandemic (Figure 1A). Concurrently, the prevalence of severe depression is also rising (Figure 1B). The prevalence was consistently higher in women than in men (Figure 1C). Among racial groups, the increasing trend was most notable in non‐Hispanic White individuals (Figure 1D). These findings provide a crucial epidemiological background for subsequent analyses.

### 3.2. Baseline Association Between Depression and Gallstone Risk

From the 2017 to 2023 NHANES cycles, we identified participants with complete gallstone questionnaire data (Figure S1). After excluding those with missing data on key covariates (including PIR, education, and BMI), 7952 adults were included in the baseline characteristic analysis (Table 1). The weighted sample represented 145,866,538 noninstitutionalized US adults. The median age of the cohort was 45 years, with a nearly equal sex distribution. Non‐Hispanic White individuals constituted the largest racial group. As presented in Table 2, the baseline health characteristics of the study population are summarized. The prevalence of depression (defined as a PHQ‐9 score ≥ 10) was 9.1%. A total of 8.6% of participants reported a prior diagnosis of gallstones. The prevalences of hypertension, hypercholesterolemia, and diabetes were 29%, 34%, and 9.2%, respectively.

**Table 1:** Population baseline data table for inclusion in the study.

Characteristic	No weighted^a^	Weighted^a^
	*N* = 7952	*N* = 145,866,538
Age	50 (34, 63)	45 (32, 60)
Sex		
Female	4102 (52%)	73,579,722 (50%)
Male	3850 (48%)	72,286,816 (50%)
Race		
Mexican American	745 (9.4%)	10,726,455 (7.4%)
Other Hispanic	735 (9.2%)	10,187,539 (7.0%)
Non‐Hispanic White	3920 (49%)	97,317,817 (67%)
Non‐Hispanic Black	1567 (20%)	14,889,677 (10%)
Other/multiracial	985 (12%)	12,745,050 (8.7%)
Education		
Less than 9th grade	247 (3.1%)	2,473,989 (1.7%)
9–11th grade	595 (7.5%)	7,487,214 (5.1%)
High school graduate	1686 (21%)	35,156,623 (24%)
Some college or AA degree	2737 (34%)	47,266,723 (32%)
College graduate or above	2687 (34%)	53,481,990 (37%)
Poverty–income ratio	2.87 (1.47, 5.00)	3.55 (1.88, 5.00)
BMI	29 (25, 34)	29 (25, 33)
Drink		
Yes	1378 (17%)	26,270,658 (18%)
No	6574 (83%)	119,595,880 (82%)
Smoke		
Yes	3435 (43%)	61,286,397 (42%)
No	4517 (57%)	84,580,141 (58%)

^a^Median (Q1, Q3); *n* (%).

**Table 2:** Population health data form for inclusion in the study.

Characteristic	No weighted^a^ *N* = 7952	Weighted^a^ *N* = 145,866,538
PHQ‐9 score		
0–9	7171 (90%)	132,547,179 (91%)
≥10	781 (9.8%)	13,319,359 (9.1%)
Gallstones		
Yes	745 (9.4%)	12,550,449 (8.6%)
No	7207 (91%)	133,316,089 (91%)
Hypertension		
Yes	2723 (34%)	42,434,342 (29%)
No	5229 (66%)	103,432,196 (71%)
High cholesterol level		
Yes	2873 (36%)	48,957,237 (34%)
No	5079 (64%)	96,909,301 (66%)
Diabetes		
Yes	905 (11%)	13,348,411 (9.2%)
No	7047 (89%)	132,518,126 (91%)

^a^Median (Q1, Q3); *n* (%).

### 3.3. Comparison of Baseline Data

Participants were stratified by gallstones status. Compared to the nongallstones group, participants with gallstones were significantly older (*p*  < 0.001), had a higher proportion of females (*p*  < 0.001), a higher BMI (*p*  < 0.001), and higher rates of smoking (*p*  < 0.001) and hypertension (*p*  < 0.001) (Table 3). With regard to high cholesterol level and diabetes, a greater proportion of subjects in the gallstone group were found to be affected by conditions.

**Table 3:** Baseline data comparison table.

Characteristic	Gallstones, *N* = 745 (8.6%)^a^	Normal, *N* = 7207 (91%)^a^	*p* ^b^
Age	55 (42, 67)	44 (32, 59)	<0.001
Sex			<0.001
Female	9,298,796 (74%)	64,280,926 (48%)	—
Male	3,251,653 (26%)	69,035,163 (52%)	—
Race			0.2
Mexican American	950,613 (7.6%)	9,775,842 (7.3%)	—
Other Hispanic	892,981 (7.1%)	9,294,557 (7.0%)	—
Non‐Hispanic White	8,808,970 (70%)	88,508,846 (66%)	—
Non‐Hispanic Black	871,093 (6.9%)	14,018,585 (11%)	—
Other/multiracial	1,026,792 (8.2%)	11,718,258 (8.8%)	—
Education			0.001
Less than 9th grade	145,658 (1.2%)	2,328,331 (1.7%)	—
9–11th grade	815,727 (6.5%)	6,671,487 (5.0%)	—
High school graduate	3,597,061 (29%)	31,559,562 (24%)	—
Some college or AA degree	4,583,091 (37%)	42,683,632 (32%)	—
College graduate or above	3,408,913 (27%)	50,073,077 (38%)	—
Poverty–income ratio	3.18 (1.72, 5.00)	3.60 (1.91, 5.00)	0.029
BMI	32 (28, 39)	28 (25, 33)	<0.001
PHQ‐9 score			0.009
0–9	10,875,651 (87%)	121,671,528 (91%)	—
≥10	1,674,798 (13%)	11,644,561 (8.7%)	—
Drink			0.042
Yes	1,632,539 (13%)	24,638,119 (18%)	—
No	10,917,910 (87%)	108,677,970 (82%)	—
Smoke			<0.001
Yes	6,424,643 (51%)	54,861,754 (41%)	—
No	6,125,806 (49%)	78,454,335 (59%)	—
Hypertension			<0.001
Yes	5,717,709 (46%)	36,716,633 (28%)	—
No	6,832,741 (54%)	96,599,455 (72%)	—
High cholesterol level			<0.001
Yes	5,788,782 (46%)	43,168,455 (32%)	—
No	6,761,667 (54%)	90,147,633 (68%)	—
Diabetes			<0.001
Yes	2,047,737 (16%)	11,300,675 (8.5%)	—
No	10,502,712 (84%)	122,015,414 (92%)	—

^a^Median (Q1, Q3); *n* (%).

^b^Design‐based Kruskal–Wallis test; Pearson’s *X* ^2^: Rao and Scott adjustment.

### 3.4. Regression Analysis

The unadjusted analysis revealed a significant positive association between depression and gallstones (OR = 1.61, 95% CI: 1.13–2.29). This association was attenuated upon successive adjustment for covariates. In Model 1, adjusted for demographic factors (age, sex, race, education, and PIR), the OR decreased to 1.54 (95% CI: 1.02–2.32). Further adjustment for lifestyle factors (BMI, smoking, and alcohol use) in Model 2 yielded an OR of 1.36 (95% CI: 0.89–2.09). In the fully adjusted Model 3, which additionally included comorbidities (diabetes, hypercholesterolemia, and hypertension), the association was further attenuated (OR = 1.31, 95% CI: 0.84–2.02). The observed trend across models suggests that depression is a potential risk factor for gallstones, although the association was no longer statistically significant after comprehensive adjustment (Table 4).

**Table 4:** Regression analysis results.

Variable	OR	CI	*p*‐Value
Depression	1.61	1.13–2.29	0.010
Model1‐Depression + age + sex + race + education + PIR	1.54	1.02–2.32	0.041
Model2–Model1 + BMI + smoke + drink	1.36	0.89–2.09	0.152
Model3–Model2 + Hypertension + diabetes + high cholesterol	1.31	0.84–2.02	0.219

### 3.5. Dose–Response Relationship

The analysis of the PHQ‐9 score as a categorical variable (none, mild, moderate–severe, and severe) revealed a significant increase in the risk of gallstones with increasing depression severity. This finding demonstrates a clear dose–response relationship and provides further robust support for a causal association (Table 5). Multivariate regression analysis revealed this trend persisted despite a *p*‐value exceeding 0.05 (Tables S2 and S3). When the PHQ‐9 score was analyzed as a continuous variable, univariate regression revealed that higher PHQ‐9 scores were associated with an increased likelihood of gallbladder stones.

**Table 5:** Dose–response relationship results.

Variable	OR	95% CI	*p*‐Value
Categorical variable			
0–9	—	—	—
10–14	1.48	0.93, 2.36	0.10
15–19	1.67	0.98, 2.84	0.058
20–27	2.40	1.22, 4.72	0.013
Continuous variable			
PHQ9‐sum	1.05	1.03, 1.06	<0.001

### 3.6. Mediation Effect Analysis

**Figure 2:**
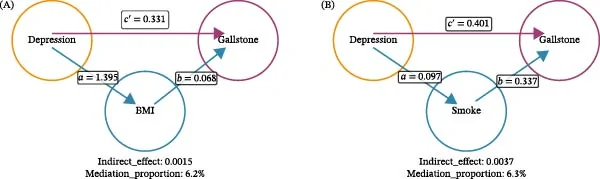
Trends in depression incidence in the United States. (A) Results of the BMI intermediary analysis. (B) Results of the smoke intermediary analysis.

In light of the findings from the multiple regression analysis, smoking was selected as the mediator from the range of lifestyle factors, and a mediation model was constructed with depression as the independent variable and gallstones as the dependent variable. The findings indicated that smoking accounted for 6.3% of the total effect (Figure 2A). The selection of BMI as the mediator for metabolic factors was further refined. The analysis indicated that BMI mediated ~6% of the total effect, suggesting that obesity plays a role in the process whereby depression leads to gallstones (Figure 2B).

## 4. Discussion

Depressive disorder and gallstone disease represent growing and significant contributors to the global burden of disease [22, 23]. Our findings are consistent with a limited number of prior observational studies [17], yet through a larger, nationally representative sample and more rigorous statistical adjustments, this study provides higher‐quality evidence. Based on the NHANES data, we report three principal findings. First, the prevalence of depression among US adults has shown a fluctuating but overall increasing trend over the past two decades, underscoring its role as a major public health concern. Concurrently, from 1989 to 2022, the prevalence of both mild‐to‐severe and moderate‐to‐severe depression among children and adolescents aged ≤18 years increased significantly over time (*p* = 0.002 and *p* = 0.034, respectively) [24]. At the global level, depression affects ~250 million people, and although prevalence rates vary across countries, both the absolute number of cases and the overall depression rate have continued to rise [25].

Second, depression was significantly associated with higher odds of gallstone disease, and there was evidence of a positive dose–response association. Consistent findings have also been reported in other relevant studies. Using Mendelian randomization analysis, Wang et al. [26] identified a causal link between genetically predicted depression and an increased risk of cholelithiasis (OR = 2.06). By analyzing NHANES data collected from 2017 to 2020, Wang’s research team further demonstrated a significant association between depressive symptoms and gallstone disease (OR = 2.001) [27]. However, in the fully adjusted model (Model 3), the association lost significance (OR = 1.31, 95% CI: 0.84–2.02), though the positive trend persisted. This may be due to insufficient power, overadjustment, or residual confounding. Nonetheless, the dose–response pattern across depression severity levels still suggests a possible link, warranting further research with larger samples, refined covariates, or alternative methods.

Third, this association appeared to be partially mediated by depression‐related obesity and smoking behavior. Notably, mediation analysis revealed that smoking and BMI accounted for merely 6.3% and 6% of the total effect, respectively. Multiple other factors may be implicated in this relationship. For instance, pro‐inflammatory diets can induce a state of chronic systemic inflammation [28], which in turn facilitates gallstone formation [16]. Meanwhile, the gut–brain axis, regulated by gut microbiota, modulates mental health [29] and further affects bile acid homeostasis via regulation of the HPA axis [12].

Notably, similar to the findings of some observational studies [30], our stratified analyses indicated that the association between depression and gallstone disease was more pronounced in women. This observation may be partly explained by potential interactions with sex hormones, which have been implicated in both mood regulation and cholesterol metabolism. Estradiol disrupts bile acid metabolism, increases bile cholesterol saturation, and induces gallstone formation [31]. Mechanistically, estrogen receptor alpha (ER*α*) can directly bind to core genes responsible for bile acid synthesis (e.g., Cyp7a1), demonstrating a pivotal role of estrogen signaling in the maintenance of bile acid homeostasis [32]. Additionally, early growth response 1 (Egr1) acts as a key mediator of estrogen‐modulated emotional behaviors, which exerts specific anxiolytic and antidepressant effects in the female brain but exhibits no comparable regulatory function in males [33]. Furthermore, Ni et al. [34] established a novel molecular axis underlying estrogen‐mediated emotional regulation, and identified progranulin (PGRN) in hippocampal neurons as the critical downstream effector that mediates the protective effects of estrogen against emotional impairment. Additionally, individuals with gallstone disease tended to be older and had a higher prevalence of comorbidities such as hypertension, hypercholesterolemia, and diabetes. These findings highlight the complex interplay between mental health, metabolic factors, and hepatobiliary disease [23, 35].

Metabolic disturbances arising from the excessive activation of the hypothalamic–pituitary–adrenal axis in people with depression have been identified as a risk factor for gallstone disease [36, 37]. Mediation analysis further revealed that both smoking and BMI served as significant mediators in the pathway from depression to gallstone formation. This suggests that depression may potentially contribute to gallstone disease not only through direct psychobiological mechanisms but also indirectly by promoting unhealthy behaviors and weight gain. Importantly, given that smoking and BMI only account for a fraction of the total mediating effect, interventions targeting these two factors alone may exert limited protective effects against gallstone development. Therefore, concurrent management of depressive symptoms remains essential, and additional unrecognized intermediate pathways warrant further investigation.

From a clinical perspective, these results imply that physicians should maintain a higher index of suspicion for gallstone disease in patients with depression, particularly among women, younger individuals, and those with concurrent obesity or metabolic abnormalities. In selected high‐risk cases, abdominal ultrasonography screening or targeted health education regarding gallstone symptoms may be warranted [38]. Moreover, the management of depression should extend beyond psychiatric symptom control to include integrated lifestyle interventions—such as dietary counseling, promotion of physical activity, and weight management programs [39]. Such holistic approaches may not only improve mental health outcomes but also directly mitigate the risk of developing gallstones.

Despite the strengths of this study, several limitations should be acknowledged. First, as NHANES data are primarily cross‐sectional, it is difficult to definitively establish the temporal sequence between depression and gallstone diagnosis. Symptomatic gallstone disease may trigger physical discomfort, pain and impaired quality of life, which subsequently increases the risk of depressive symptoms. Second, gallstone status was based on self‐report, which is susceptible to both underreporting and misclassification. Third, despite adjusting for a wide range of potential confounders, unmeasured variables such as detailed dietary habits, genetic predisposition or specific medication use may still contribute to residual confounding. Fourth, mediation analysis based on cross‐sectional data yields exploratory findings, which cannot confirm causal mechanisms given the lack of temporality and potential residual confounding.

In conclusion, this study reinforces the link between depression and gallstone disease, identifies potential mediating pathways, and underscores the importance of comprehensive and preventive healthcare strategies in mentally vulnerable populations.

## 5. Conclusion

This study demonstrates a rising prevalence of depression among US adults and provides evidence for an association between depression and gallstone disease. The positive dose–response relationship across depressive symptom grades reinforces this link, while smoking and BMI partially mediate the association. Clinically, integrated mental and metabolic health management should be considered for individuals with depression to address potential biliary complications. Owing to the cross‐sectional nature of NHANES data, causal inference cannot be drawn. Longitudinal research is necessary to elucidate the temporal sequence and biological mechanisms underlying the relationship between depression and gallstone disease.

## Author Contributions

Research idea and design: Enyang He and Bei Li. Data acquisition: Enyang He, Yu Teng, and Yaoqun Wang. Data analysis: Enyang He, Guilin Nie, Di Zeng, Jiaqi Yang, and Xinming Li. Manuscript writing: Enyang He, Yaoqun Wang, Qingyan Kong, Fei Liu, Geng Liu, and Guilin Nie. Reviewing: Bei Li and Nansheng Cheng.

## Funding

This work was supported by the 2026 Open Research Fund of Key Laboratory for Digital Innovation of Tianfu Culture, Sichuan Provincial Department of Culture and Tourism.

## Ethics Statement

The study was based on open‐source data from NHANES databases. Ethical approval has been provided for the patients involved in these databases. Therefore, there are no ethical issues with this article.

## Consent

The authors have nothing to report.

## Conflicts of Interest

The authors declare no conflicts of interest.

## Supporting Information

Additional supporting information can be found online in the Supporting Information section.

## Supporting information


**Supporting Information** Table S1: Presents the specific items of the Patient Health Questionnaire (PHQ‐9). Figure S1: Illustrates the analysis workflow of the 2017–2023 NHANES cycles. Tables S2 and S3: Provide the results of multivariate regression analyses for the dose–response relationship.

## Data Availability

The data that support the findings of this study are openly available in NHANES at https://www.cdc.gov/.
