# Supplementary material for: Trends in Depression and Its Association With Gallstones: Evidence From NHANES With Exploration of Mediating Pathways
Source: Depress Anxiety. 2026 Sep 22;2026:1800832. doi: 10.1155/da/1800832 (PMC13595807; doi:10.1155/da/1800832)
Supplement: Supplementary file 1 [file DA-2026-1800832-s001.docx]

**Supplementary Figure S1**

Supplementary Figure S1: Research participant screening process diagram.

**Supplementary Table1**

| **Patient Health Questionnaire (PHQ-9)** | |
| --- | --- |
|  | Value |
| Have little interest in doing things |  |
| Feeling down, depressed, or hopeless |  |
| Trouble sleeping or sleeping too much |  |
| Feeling tired or having little energy |  |
| Poor appetite or overeating |  |
| Feeling bad about yourself |  |
| Trouble concentrating on things |  |
| Moving or speaking slowly or too fast |  |
| Thought you would be better off dead |  |
| Sum |  |

| Value Description | Value |
| --- | --- |
| Not at all | 0 |
| Several days | 1 |
| More than half the days | 2 |
| Nearly every day | 3 |

**Supplementary Table2**

| **Characteristic** | **OR***^1^* | **95% CI***^1^* | **p-value** |
| --- | --- | --- | --- |
| PHQ9_category |  |  |  |
| 0-9 | — | — |  |
| 10-14 | 1.22 | 0.69, 2.16 | 0.5 |
| 15-19 | 1.38 | 0.81, 2.37 | 0.2 |
| 20-27 | 1.73 | 0.79, 3.82 | 0.2 |
| Age | 1.03 | 1.02, 1.04 | <0.001 |
| Sex |  |  |  |
| Female | — | — |  |
| Male | 0.32 | 0.25, 0.42 | <0.001 |
| Race |  |  |  |
| Mexican American | — | — |  |
| Other Hispanic | 0.89 | 0.63, 1.26 | 0.5 |
| Non-Hispanic White | 0.73 | 0.56, 0.95 | 0.021 |
| Non-Hispanic Black | 0.39 | 0.27, 0.57 | <0.001 |
| Other/multiracial | 0.86 | 0.54, 1.38 | 0.5 |
| Education |  |  |  |
| Less than 9th grade | — | — |  |
| 9-11th grade | 1.96 | 1.06, 3.66 | 0.035 |
| High school graduate | 1.86 | 1.02, 3.37 | 0.043 |
| Some college or AA degree | 1.77 | 1.04, 3.03 | 0.038 |
| College graduate or above | 1.43 | 0.77, 2.63 | 0.2 |
| Poverty-income Ratio | 0.98 | 0.91, 1.06 | 0.6 |
| BMI | 1.07 | 1.05, 1.08 | <0.001 |
| Drink |  |  |  |
| No | — | — |  |
| Yes | 0.89 | 0.59, 1.34 | 0.6 |
| Smoke |  |  |  |
| No | — | — |  |
| Yes | 1.41 | 1.10, 1.80 | 0.008 |
| Hypertension |  |  |  |
| No | — | — |  |
| Yes | 1.22 | 0.90, 1.67 | 0.2 |
| High cholesterol level |  |  |  |
| No | — | — |  |
| Yes | 1.13 | 0.87, 1.47 | 0.3 |
| Diabetes |  |  |  |
| No | — | — |  |
| Yes | 1.12 | 0.78, 1.60 | 0.5 |
| *^1^* OR = Odds Ratio, CI = Confidence Interval | | | |

**Supplementary Table3**

| **Characteristic** | **OR***^1^* | **95% CI***^1^* | **p-value** |
| --- | --- | --- | --- |
| PHQ9-Sum | 1.02 | 1.00, 1.04 | 0.093 |
| Age | 1.03 | 1.02, 1.04 | <0.001 |
| Sex |  |  |  |
| Female | — | — |  |
| Male | 0.33 | 0.26, 0.42 | <0.001 |
| Race |  |  |  |
| Mexican American | — | — |  |
| Other Hispanic | 0.89 | 0.63, 1.26 | 0.5 |
| Non-Hispanic White | 0.72 | 0.55, 0.95 | 0.020 |
| Non-Hispanic Black | 0.39 | 0.27, 0.57 | <0.001 |
| Other/multiracial | 0.86 | 0.54, 1.38 | 0.5 |
| Education |  |  |  |
| Less than 9th grade | — | — |  |
| 9-11th grade | 1.98 | 1.07, 3.68 | 0.032 |
| High school graduate | 1.87 | 1.03, 3.39 | 0.040 |
| Some college or AA degree | 1.79 | 1.05, 3.04 | 0.034 |
| College graduate or above | 1.44 | 0.78, 2.64 | 0.2 |
| Poverty-income Ratio | 0.98 | 0.92, 1.06 | 0.6 |
| BMI | 1.07 | 1.05, 1.08 | <0.001 |
| Drink |  |  |  |
| No | — | — |  |
| Yes | 0.89 | 0.59, 1.34 | 0.6 |
| Smoke |  |  |  |
| No | — | — |  |
| Yes | 1.40 | 1.10, 1.79 | 0.009 |
| Hypertension |  |  |  |
| No | — | — |  |
| Yes | 1.22 | 0.90, 1.67 | 0.2 |
| Cholesterol |  |  |  |
| No | — | — |  |
| Yes | 1.13 | 0.87, 1.46 | 0.4 |
| Diabetes |  |  |  |
| No | — | — |  |
| Yes | 1.12 | 0.79, 1.60 | 0.5 |
| *^1^* OR = Odds Ratio, CI = Confidence Interval | | | |
